# Implementation of a Dedicated Intake Team Reduces Time to Massed PTSD Treatment

**DOI:** 10.1007/s11414-024-09920-4

**Published:** 2024-12-17

**Authors:** Jennifer A. Coleman, Brianna Werner, Brian J. Klassen, Dale L. Smith, Uddyalok Banerjee, Philip Held

**Affiliations:** 1 Department of Psychiatry, Rush University Medical Center, 1645 W. Jackson Blvd., Suite 602, Chicago, IL 60612 USA; 2 Department of Psychiatry, University of Illinois at Chicago, Chicago, IL USA

## Abstract

The Institute of Medicine (2001) describes quality health care as safe, effective, patient-centered, efficient, equitable, and timely. Although this definition highlights the necessity of continuous program evaluation to ensure that these goals are being addressed, there is a notable lack of industry-wide standards and benchmarks, and many clinical programs lack the ability to continually and rigorously evaluate their own performance with data. This might be particularly true in the case of ensuring service members and veterans with posttraumatic stress disorder (PTSD) obtain treatment, as several systemic barriers exist, such as long wait times and lack of equitable treatment for individuals with minoritized identities. The current study examines the impact of a clinic-wide intake redesign for a massed PTSD treatment program to shift the intake process to a small, dedicated team rather than a responsibility shared across all clinicians. The redesign led to significantly shorter wait times for treatment and reduced some types of pre-treatment dropout. On average, patients received an acceptance/rejection decision 1 week sooner, attended the program almost 2 months sooner, and saw a roughly 60% reduction in the odds of dropout at the point of receiving an acceptance/rejection decision. Some disparities in wait times for those who were not partnered, women, and individuals who financially supported more family members remained after the redesign. Results are discussed in light of the importance of continuous program evaluation to address IOM’s holistic definition of quality health care.

## Introduction

The Institute of Medicine (IOM)^1^ describes quality health care to be safe, effective, patient-centered, efficient, equitable, and timely. Although holistic and aspirational, system-wide implementation of these standards has been difficult, because there is sometimes a lack of national standards for health care organizations to compare themselves to.^2^ as well as significant variability on key performance metrics like wait times.^3^ Engaging in systematic identification and removal of barriers has been discussed as a major way for health care organizations to reduce disparities.^4^

Such health disparities may present as longer wait times which are associated with worse patient satisfaction, retention, and outcomes.^5^–^10^ Hence, there has been a focus on designing systems that improve the time to receive care.^11^ The military community is interested in time-to-care metrics, especially as it relates to posttraumatic stress disorder (PTSD) and other trauma-related concerns.^12^,^13^ Untreated PTSD has been shown to increase the risk for a variety of other mental and medical comorbidities, including risk for suicide and cardiovascular issues, among many others.^14^,^15^ To reduce the overall burden of untreated PTSD, it is critical for clinics that provide PTSD treatment to continuously evaluate how long it takes for patients to receive treatment and evaluate strategies to improve this metric.

Another metric of health disparities to consider is that access to care is not equitable depending on gender, race, ethnicity, and socioeconomic status.^16^,^17^ In veteran populations, African American/Black and Hispanic veterans are less likely to have adequate medication trials, and African American/Black veterans are also less likely to engage in adequate sessions of psychotherapy thus reducing opportunites for effective treatment.^18^ Research has also found that wait time for medical care worsened from pre- to post-COVID for many African American/Black and Hispanic veterans.^19^ Thus, in addition to examining overall time to care, strategies to reduce health disparities require that access is equitable across different patient populations.

An important aspect of reducing health disparities is providing equitable access to evidence-based mental health care. Trauma-focused cognitive behavioral therapies, such as Cognitive Processing Therapy^20^ and Prolonged Exposure,^21^ have amassed considerable evidence for their efficacy and effectiveness, and are recommended as first-line interventions for the treatment of PTSD.^22^ These treatments are widely disseminated in large health care systems, such as the Department of Veterans Affairs, and also provide the foundation for many PTSD specialty clinics. Some PTSD specialty clinics have successfully delivered these evidence-based treatments in a massed format by delivering multiple sessions per day, permitting patients to finish an entire course of treatment in two weeks or less.^23^–^28^ Furthermore, many of these specialty clinics also require their patients to attend in-person programming which necessitates patients to be physically healthy enough to independently complete activities of daily living to be accepted for programming as well as travel, sometimes out of state, to complete programming. Yet, the process of getting accepted into a specialty PTSD treatment (as opposed to traditional weekly outpatient care), including the time it takes to receive care, is relatively understudied.

In both mental health specialty clinics and non-specialty clinics, it is common for mental health providers across an organization to have multiple roles. For example, mental health providers may not only deliver evidence-based psychotherapies, but may also conduct intake evaluations, obtain and review medical records, and staff cases to an interdisciplinary team, etc. Having to “wear multiple hats” increases their role complexity and can reduce role clarity. Increased role clarity among providers, however, can result in higher quality of care for patients and reduce burnout for providers.^29^–^31^ Furthermore, systemic changes such as implementing a focused intake team and including a Patient Care Navigator (PCN; i.e., a non-clinically licensed staff member who offers patients support and resources and registers them for services) role have proven to be successful in reducing wait times for psychotherapy and improving client-centered support at academic medical centers.^32^,^33^

The goal of the current project was to evaluate a system-wide clinic redesign that shifted responsibility for clinical intakes from all clinicians in the organization to a small, dedicated intake team. With the overall goal to reduce time to treatment for patients as well as to increase role clarity for providers, designated intake clinicians and PCNs were introduced. A previous review of patient navigation services found that PCNs are integral in (1) reducing barriers to mental health care (providing psychoeducation about mental illness, the mental health system, connecting patients and providers); (2) providing client-centered support, individual needs assessment including building a consistent, ongoing relationship, and advocacy; and (3) providing integrated care and assisting with transitions in care (e.g., preparing to enter the massed treatment program and facilitating aftercare, as needed).^33^ Based on prior literature, the authors hypothesized that the implementation of a dedicated intake team would reduce time to care and pre-treatment dropout rates in the PTSD specialty clinic. Furthermore, exploratory analyses were conducted to examine whether a dedicated intake team would reduce health disparities for patients with minoritized identities based on reported sex, race, and ethnicity.^25^

## Methods

### Sample Description

Data were examined for service members and veterans (*N* = 1010) who registered and attended the Intensive Treatment Program (ITP) between January 2021 and September 2023, with additional data pulled for individuals who attended the ITP within this time frame but who registered or completed an intake prior to January 2021. This time frame was chosen to achieve roughly equal samples pre- and post-intake team. The average number of intake appointments offered by the clinic per month remained similar pre-intake and post-intake team, with roughly ten novel intake appointments available per week. Exclusions (*n* = 155) encompassed individuals who transferred from the outpatient clinic to ITP (*n* = 25), repeat ITP participants (*n* = 20; only first attendance data were included), intakes conducted by non-intake clinicians post-intake team formation (*n* = 109), and one outlier. Individuals were on average 43.38 years old (*SD* = 10.13), 59% identified as male, 50% as married or partnered, 83% as non-Hispanic/Latino, and 64% identified as white. Additional sample descriptives can be found in Table 1.

**Table 1:** Demographic characteristics

	Total sample (*N* = 855)	Pre-intake team (*n* = 471)	Post-intake team (*n* = 384)
Age (years; *M*, *SD*)	43.38 (10.13)	42.93 (9.85)	43.92 (10.44)
Sex (*n*, %)			
Male	502 (58.6%)	271 (57.5%)	231 (60.2%)
Race (*n*, %)			
American Indian/Alaska Native	13 (1.5%)	9 (1.9%)	4 (1.0%)
Asian	26 (3.0%)	12 (2.6%)	14 (3.7%)
Black/African American	196 (22.9%)	102 (21.7%)	94 (24.5%)
Native Hawaiian/Pacific Islander	11 (1.3%)	7 (1.5%)	4 (1.0%)
Other	60 (7.0%)	23 (4.9%)	37 (9.6%)
White	549 (64.2%)	318 (67.5%)	231 (60.2%)
Ethnicity (*n*, %)			
Not Hispanic/Latino	710 (82.8%)	393 (83.4%)	316 (82.3%)
Marital status (*n*, %)			
Married or partnered	425 (49.6%)	231 (49.0%)	194 (50.5%)

### Overview of Clinic

The study was conducted at the Road Home Program at Rush University Medical Center, a philanthropically funded specialty mental health clinic serving service members, veterans, and their families at no out-of-pocket cost. Approximately 25% of individuals are referred to the program from the Wounded Warrior Project; other individuals hear about the program by word of mouth or through outreach programming. The program accepts service members and veterans with PTSD related primarily to combat trauma or military sexual trauma regardless of discharge status or location of residence, thus making the program accessible to individuals from all over the United States and its territories even though the program is in Chicago, IL. Individuals were required to stay in apartment-style housing near the treatment facility for the duration of the ITP. Cost for travel (airfare or milage reimbursement), housing, food, and all clinical services are covered by the program.

For this study, the authors examined time to care and equitable access to the ITP. While in the ITP, patients received daily CPT in combination with adjunctive services (see Held et al., 2022, for a more detailed treatment description and program outcomes) according to a pre-determined schedule. Given the ITP is a higher level of care than a traditional outpatient program, yet a lower level of care than a residential program and staffed with primarily mental health therapists without access to full medical services, the intake process for the ITP was necessarily more detailed and time consuming for patients compared to the intake process for standard outpatient care.

### Intake Process

All interested service members and veterans were required to complete a standardized intake assessment to determine the goodness of fit for the ITP. The ITP intake process consisted of roughly seven steps: (1) a 30-min registration call with a PCN, including setting up electronic messaging capabilities in the electronic medical record system and completing releases of information to obtain medical records; (2) a 90-min biopsychosocial intake assessment with a clinician; (3) symptom measure completion via a common electronic survey platform; (4) a structured clinical diagnostic interview with a clinician to rule out the need for a higher level of care; (5) the Clinician-Administered PTSD Scale for DSM-5^34^ assessment to confirm a PTSD diagnosis; (6) medical record review; and (7) an interdisciplinary staff meeting where acceptance/rejection decisions were made. If a patient was not accepted for care, they were referred for appropriate services. Roughly 91% of patients who completed the intake were accepted into the ITP, at which time a PCN maintained contact with the patient at intervals until they attended the program. Intakes were primarily conducted virtually (via video or phone) because most patients (86%) who attended the program did not reside in Illinois, although local patients had the option to attend in-person intake appointments.

Prior to November 2021, intakes for the ITP were conducted by various clinical providers with a range of clinical degrees (social work externs, social workers, mental health counselors, psychology postdoctoral fellows, and psychologists). In addition to delivering psychotherapy, these providers were also required to conduct ITP intakes as outlined above and carry out any associated case management duties (including obtaining and reviewing medical records, etc.). Before implementing the dedicated intake team, the clinic had no consistent set of norms regarding typical intake timelines.

The intake team was formed in November 2021 and consisted of one bachelor’s level PCN, two master’s level clinicians, and one PhD-level manager. The implementation of an intake team aimed to develop a streamlined intake process, increase staff adherence to the intake process, reduce the burden and case management responsibilities on the ITP clinical team, and reduce wait time for intake evaluations, acceptance/rejection decisions, and overall ITP start times. The primary aim of the PCN role was to provide greater continuity of care for patients going through the intake process, as the PCN is the first and last point of contact for patients attending the program. The role of the PCN was to register patients, assist with case management needs (e.g., obtain medical records, assess for financial barriers, etc.), conduct engagement calls, assess motivation for attending programming prior to the ITP, track clinical recommendations (e.g., substance use reduction, medication compliance, establishing outpatient therapy), update ITP staff when patients cannot attend programming, conduct safety planning related to substance use reduction and suicidal ideation, and complete two follow-up calls once a patient completes the ITP.

In contrast, the role of an intake clinician was to conduct clinical intake assessments, review medical records and symptom measures, consult with the medical team about patients’ appropriateness for services given medical conditions, and present each case at an interdisciplinary staff meeting to determine eligibility. Intake clinicians were given guidelines to complete all intake appointments within a week (unless patients were unavailable), staff cases to obtain an acceptance/rejection decision within 3 to 4 weeks, and request any patients who could not attend an ITP within 3 to 4 months to schedule an intake at a time closer to when they could attend the ITP. An intake team manager (psychologist) oversaw the team, consulted on cases, ensured a systematic process was followed, reviewed the status of intakes to confirm they were moving forward efficiently, and co-led interdisciplinary staffing meetings to assess fit for ITP. After the implementation of the intake team, a small percentage of intakes were still completed by a few non-intake team clinicians during times of low clinical workload and by postdoctoral fellows for training purposes. These data were removed from analyses in the post-intake team group.

### Variables Assessed

*Demographic characteristics.* Service members and veterans self-reported various demographic characteristics during their registration. Information about age, sex (male vs female), race (white vs other), ethnicity (Hispanic/Latino vs non-Hispanic/Latino), employment status (retired and unemployed vs other), number of family members supported, referral source, and marital status (married or partnered vs other) was included in analyses to determine the potential impact on wait times.

*Time to receive services.* Time to receive services in number of days was compared pre-intake team (prior to October 31, 2021) to post-intake team (after November 1, 2021). Four program milestones were used for analyses: (1) date of registration for services, (2) date of first completed ITP intake appointment, (3) date of acceptance/rejection decision, and (4) ITP start date. Analyses examined (1) time from registration to first ITP intake, (2) time from registration to acceptance/rejection decision, and (3) time from registration to ITP start date.

*Dropout.* Dropout was defined as a service member or veteran not progressing to the next stage in the intake process for any given reason. For statistical analyses, at the second and third program milestones, the authors excluded cases who had dropped out during previous program milestones. Examples of dropout may include an individual registering but not completing an intake, an individual completing an intake but being lost to contact before a decision was made, or an individual completing their registration and intake but later choosing not to attend the ITP.

### Statistical Analysis

Following descriptive analyses of time to receive services and dropout, the authors explored predictors of time to various program milestones described above and dropout. Multivariable linear regression was used to explore predictors of the number of days to receive each service, and multivariable logistic regression was used to explore predictors of dropout at each point. Individuals were excluded if they completed multiple ITPs and their second ITP fell within the time frame of this study given that the intake process may have been different from novel participants. Patients who completed outpatient services with the clinic prior to the ITP were also excluded given the same rationale. Lastly, this study excluded any cases in which intakes were completed by other (non-intake team) clinical staff which occurred after the implementation of the intake team.

Separate models for each intake process segment were examined due to specific interest in predictors of events and expectations that predictors may differ across program milestones. Along with intake team status, all models included—and thus adjusted for—age, sex, race, ethnicity, marital status, referral source, number of family members financially supported, and whether patients were currently employed or students.^1^ Multicollinearity was examined via variance inflation factor (VIF) analysis, though no VIFs above 2 were identified. Unstandardized slopes and odds ratios were reported for ease of interpretation. All analyses were conducted in Python version 3.8.1.

## Results

Prior to the implementation of the intake team, 34 unique clinicians conducted intakes. After the implementation of the intake team, four unique clinicians provided intakes (note: one provider left and was replaced). Across the entire sample of service members and veterans, the average time from registration to the first ITP intake appointment attended was 9.88 days (*SD* = 15.41), the average time from registration to acceptance/rejection decision was 44.24 days (*SD* = 43.44), and the average time from registration to ITP start date was 109.14 days (*SD* = 94.14; see Table 2).

**Table 2:** Wait time to care in days and dropout rates

	Total sample	Pre-intake team	Post-intake team
Registration to intake *(SD)*	9.88 (15.41)	10.44 (19.73)	9.22 (7.94)
Registration to decision *(SD)*	44.24 (43.44)	44.28 (36.38)	36.49 (24.87)
Registration to ITP start *(SD)*	109.14 (94.14)	140.90 (124.33)	82.64 (42.97)
Dropout before intake (*n*, %)	104 (855, 12.06)	69 (471, 14.65)	35 (384, 9.11)
Dropout before decision (*n*, %)	100 (707, 14.14)	75 (381, 19.69)	25 (326, 7.67)
Dropout before ITP start (*n*, %)	60 (504, 11.90)	34 (236, 14.41)	26 (268, 9.70)

Multivariable linear regression analyses indicated that the implementation of the intake team was not significantly associated with number of days to an intake appointment (*b* = − 1.18, *p* = 0.30), but was associated with fewer days to ITP acceptance (*b* = − 14.11, *p* < 0.001), and ITP start date (*b* = − 52.41, *p* < 0.001). When adjusting for other variables, the change in number of days to intake from the pre-intake team of 10.44 days (*SD* = 19.73) to the post-intake team of 9.22 days (*SD* = 7.94) was not significant. The number of days to acceptance/rejection decision dropped from 44.28 days (*SD* = 36.38) to 36.49 days (*SD* = 24.87), and the number of days to ITP start decreased from 140.90 days (*SD* = 124.33) to 82.64 days (*SD* = 42.97), both were significant in adjusted models. Being female was associated with more days to acceptance/rejection decision (*b* = 8.58, *p* = 0.02) and days to ITP start (*b* = 18.41, *p* = 0.04), and supporting more family members was associated with more days to ITP start (*b* = 8.67, *p* = 0.001). No other variables were significantly associated with days to intake appointment or days to acceptance/rejection decision (see Table 3).

**Table 3:** Predictors of time to intake, decision, and program start

Predictor	Days to intake	*p*	Days to decision	*p*	Days to ITP start	*p*
	*b* (95% CI)		*b* (95% CI)		*b* (95% CI)	
Team	− 1.18 (− 3.44, − 1.07)	.303	− 14.11 (− 21.07, − 7.15)	< .001	− 52.41 (− 69.44, − 35.38)	< .001
Age	0.05 (− 0.07, 0.16)	.454	0.01 (− 0.36, 0.38)	.963	− 0.05 (− 0.96, 0.86)	.914
Sex	1.26 (− 1.05, 3.58)	.284	8.58 (1.38, 15.78)	.020	18.41 (0.92, 35.89)	.039
Race	− 0.29 (− 2.69, 2.10)	.811	3.19 (− 4.33, 10.70)	.405	7.17 (− 11.14, 25.48)	.442
Ethnicity	1.58 (− 1.44, 4.61)	.384	3.12 (− 5.90, 12.13)	.497	1.50 (− 20.58, 23.59)	.894
Marital	− 1.98 (− 4.39, 0.43)	.108	0.85 (− 6.92, 8.62)	.830	− 18.59 (− 37.83, 0.64)	.058
Referral	0.64 (− 1.65, 2.95)	.579	− 2.56 (− 9.58, 4.46)	.475	9.40 (7.63, 26.44)	.279
Family	0.19 (− 0.47, 2.21)	.564	1.29 (− 0.85, 3.43)	.236	8.67 (3.41, 13.93)	.001
Employment	− 0.15 (2.51, 2.20)	.901	− 3.60 (− 10.83, 3.64)	.329	− 10.86 (− 20.33, 6.62)	.223

Intake team implementation was associated with a significant increase in the odds of dropout before intake (OR = 2.58, *p* = 0.002), but a significant decrease in the odds of dropout at acceptance (*b* = 0.39, *p* < 0.01). No significant difference in dropout by ITP start was found based on intake team status (*b* = 0.63, *p* = 0.11). Being married or in a domestic partnership, referral from the Wounded Warrior Project, and supporting fewer family members were associated with reduced odds of dropout before intake and acceptance, but not dropout before ITP start (see Table 4). Being female was also associated with reduced odds of dropout at acceptance and ITP start, though no other demographic variable was associated with dropout across the three measured program milestones (Table 4). Notably, individuals who dropped out at each program milestone were excluded from analyses of dropout at the next program milestone, each analysis tests predictors only of dropout during that segment and relevant variables predicting dropout at earlier segments may no longer be impactful in later segments (see Table 2).

**Table 4:** Predictors of dropout in logistic regression models

Predictor	Dropout before intake	*p*	Dropout before decision	*p*	Dropout before ITP start	*p*
	*b* (95% CI)		*b* (95% CI)		*b* (95% CI)	
Team	2.58 (1.39, 4.77)	.002	0.39 (0.24, 0.64)	< .001	0.63 (0.36, 1.12)	.114
Age	0.99 (0.96, 1.02)	.379	0.98 (0.96, 1.01)	.142	0.98 (0.95, 1.02)	.323
Sex	1.15 (0.69, 1.12)	.588	0.61 (0.38, 0.99)	.047	0.49 (0.27, 0.91)	.024
Race	0.92 (0.54, 1.55)	.749	1.02 (0.63, 1.65)	.941	1.08 (0.59, 1.97)	.811
Ethnicity	1.77 (0.97, 3.25)	.065	0.55 (0.28, 1.09)	.087	1.09 (0.54, 2.20)	.818
Marital	0.48 (0.29, 0.79)	.004	0.59 (0.36, 0.94)	.028	0.54 (0.29, 1.03)	.060
Referral	0.35 (0.20, 0.59)	< .001	0.87 (0.55, 1.37)	.549	1.53 (0.87, 2.70)	.136
Family	2.23 (1.91, 2.61)	< .001	1.18 (1.06, 1.31)	.003	1.05 (0.89, 1.23)	.580
Employment	0.10 (0.54, 1.50)	.685	0.90 (0.57, 1.42)	.646	1.23 (0.70, 2.16)	.479

## Discussion

The goal of this study was to evaluate a clinic redesign that involved shifting responsibility for intakes for a PTSD ITP from all clinical staff to a dedicated intake team consisting of clinicians and PCNs. The redesign led to significantly shorter wait times for treatment and reduced some types of pre-treatment dropout. On average, patients received an acceptance/rejection decision 1 week sooner, attended the program almost 2 months sooner, and saw a roughly 60% reduction in the odds of dropout at the point of receiving an acceptance/rejection decision. Some disparities in wait times for those who were not partnered, women, and individuals who financially supported more family members remained.

The finding that a dedicated intake team and introduction of care navigation were associated with significantly shorter wait time for treatment is consistent with previous literature.^32^,^33^ Prior to the intake team, clinicians had a myriad of clinical duties. ITP clinicians generally provided 4 hours of trauma-focused psychotherapy sessions per day in addition to conducting intakes and seeing outpatients. Given the cadence of the ITPs being scheduled back-to-back, ITP clinicians often had limited availability to complete the multi-step intake process. Forming a devoted intake team allowed intake clinicians to focus on intake tasks leading to staffing cases and obtaining an acceptance/rejection decision quicker. Implementing the intake team also led to the creation of specific guidelines regarding metrics for an “efficient” intake process for the staff to follow. Implementation of the PCN role also allowed for any open ITP slots (due to a patient drop) to be filled more efficiently, and thus wait time for treatment to be reduced as the intake team manager only had to work with the small PCN team to find patients who could attend an earlier ITP instead of assessing for patient availability among over 30 different staff members. Prior to the intake team, the average intake appointment occurred roughly 10 days (including weekends) after registration, and thus the drop to roughly 9 days post-intake team was not significant. The finding that there was no significant difference in wait time pre- and post-intake team for initial appointment was unsurprising, given that scheduling intake appointments within 15 days of registration is considered best practice.^35^

The variance in wait time was considerably reduced once the intake team was established. The variance of days from registration to intake decreased by roughly 11 days, the variance in time to acceptance/rejection decision decreased by roughly 12 days, and the variance in time to ITP attendance decreased by 81 days. Reducing variance in wait time suggests that dedicated intake clinicians may follow a more uniform intake process, which may improve wait time while also possibly reducing the likelihood of disparities for select groups of patients. A standardized and effective intake process is also consistent with the IOM’s guidelines on efficient, equitable, and timely access to quality care.^1^

Despite decreases in wait time following the implementation of the intake team, there were disparities based on certain demographic characteristics. Being female was associated with longer time until obtaining a decision and beginning the ITP. Financially supporting more family members was associated with more days to ITP start. Individuals who were married or partnered, referred by an outside organization, and who financially supported fewer family members had decreased odds of dropping out before intake and before a decision. One explanation for the finding that direct referrals from partner organizations reduced dropout rates could be that these patients had received additional case management support, similar to the effect of having PCN support in improving care outcomes.^33^ Being female was also associated with lower odds of dropout at acceptance and ITP start. The finding that women faced longer wait time for receiving care is supported by the literature regarding barriers that may disproportionately affect women, such as childcare or planning time off from work, which persist beyond timely access to services.^16^,^17^

Although the finding that women faced longer wait times is consistent with the literature, the finding that individuals with minoritized racial and ethnic identities did not face longer wait time for care is contrary to the literature regarding the existence of systemic barriers to care (e.g., limited access and long wait times).^17^ African American/Black and Hispanic/Latino adults are half as likely as white adults to utilize mental health services,^17^ and African American/Black and Hispanic/Latino veterans have been found to have longer wait times for care than their white counterparts.^36^ Contrary to what this study found, research has demonstrated that patients receiving mental health care may have high rates of dropout when care is conducted through telehealth, especially for minoritized racial and ethnic patients.^37^ Potentially, the PCN team provided consistent case management support, such as identifying individuals who could benefit from referrals to organizations that provided financial support, which could in turn may have reduced barriers to attending care.

Overall, dropout rates between registration and intake across the whole sample averaged 12% suggesting that in general, the clinic has been successful in maintaining a low dropout rate. The finding that dropout was significantly higher after the intake team was implemented can be understood in the context of demographic variables. Within the current sample, post-intake team, service members, and veterans reported financially supporting fewer family members; thus, controlling for this variable led to an increase at dropout before intake. Considering demographic variables such as number of family members supported is thus an important consideration for those seeking treatment and understanding barriers to receiving care. It is also possible that with the addition of the PCN role, interested individuals more quickly obtained specific information about the ITP services available (at registration) and may have been able to identify that the ITP was not of interest or a good fit.

Although the implementation of the intake team did not significantly impact overall dropout prior to ITP attendance, it decreased dropout prior to individuals being notified of their acceptance into the ITP. Prior to the intake team, roughly 20% of individuals dropped out before the decision, whereas roughly only 8% dropped out at the same time point after the implementation of the intake team. It should be noted that although the time from registration to attending an ITP significantly decreased with an intake team, dropout at ITP attendance did not, suggesting that even with a reduction of wait time by almost 2 months for ITP attendance, dropout rates (and attendance rates) were relatively consistent at this stage. The period between intake and program start is critical, as many patients may choose to not attend treatment even after intake completion because of PTSD-related avoidance, fluctuating motivation, or other logistical and practical barriers. Reducing dropout at this stage is paramount, as substantial staff and patient resources and time have already been invested. Thus, the finding that a dedicated intake team reduced dropout at any stage is notable. Moreover, a more timely notification of acceptance into the ITP may have also increased motivation and thus decreased the likelihood of dropout following an intake, although this study was unable to specifically test these propositions due to the available data. These findings align with a review of factors associated with utilization of PTSD services which suggest that connecting individuals seeking primary care with care navigation quickly increased engagement in PTSD services.^37^

### Limitations

Limitations of the present study include limited generalizability to other groups or other types of mental health clinics and other mental health concerns. Specifically, the current study evaluated the impact of a clinic redesign in a very specific context: a PTSD ITP for veterans and service members. Future research should explore if these findings are consistent across traditional outpatient clinics and non-PTSD-focused populations. Given the dearth of literature and reporting of wait times for access to mental health care nationwide, this study was also unable to compare this clinic’s wait times to that of a national average. Furthermore, these data were collected in the context of an active clinical program, and individuals were not randomly assigned to an intake team or not. Thus, while the study may be ecologically valid, the study cannot account for historical effects or other variables that may impact the findings and cannot demonstrate causal relationships between the intake team and improved time to services. Furthermore, past research has shown that veterans with mild-moderate PTSD symptoms (compared to those with severe PTSD symptoms) may be less likely to connect to care which may contribute to the value of PCN services in increasing initial connection to mental health care in settings such as primary care offices.^37^ The current study did not assess the relationship between PTSD severity and dropout and was unable to assess barriers to those who never initiated services.

The present study highlights the importance of continually and rigorously assessing clinic processes in mental health programs, as recommended by the Institute of Medicine.^2^ As a result of the present study, the authors were able to identify ways for service members and veterans to receive intensive treatment more than 2 months sooner and reduce pre-treatment dropout. Doing so enabled service members and veterans to access effective treatment and reduces the risk for common life-threatening comorbidities of PTSD. Future research should expand on factors that encourage treatment attendance for patients who had previously dropped out from the intake process as well as the impact of procedural changes, such as the ability to make program acceptance/rejection decisions quickly as sufficient information becomes available rather than waiting for a scheduled staffing meeting. Considering the treatment gains maintained up to a year after completion of massed evidence-based PTSD treatment, the implementation of an intake team is a strong step in increased access to this care.^25^,^38^

Given the limitations of this study, there is a wealth of additional research that could enhance understanding of the benefits or disadvantages to developing a dedicated intake team. The authors’ finding that wait time is impacted by gender and financial responsibilities suggests that future research could study specific reasons for the identified differences in time to receiving care and how to best make appropriate adjustments to the intake process to facilitate equitable access for all individuals. Given this study did not explore clinician’s experience, future research could assess the effects of burnout on this type of redesign. Given past research has shown a decreased likelihood of racial/ethnic minority service members and veterans to engage in PTSD treatment, future research should attend to factors specific to those who are disproportionately impacted by system barriers as well as further research exploring why clinics such as this one did not have such barriers.^37^ Future research should also explore how decreased wait time or dropout rates could reduce health disparities based on other demographic variables such as gender identity, sexual orientation, branch of military service, or active duty versus veteran status.

## Implications for Behavioral Health

Although the Institute of Medicine ^1^ describes quality health care as safe, effective, patient-centered, efficient, equitable, and timely, the reality is that many clinics and service providers fall short of this ideal. However, a major limitation of the IOM’s definition is that there are no metrics to what is considered “efficient” or “timely care.” Additional policy should consider concrete definitions of these metrics for various types of mental health care, especially for specialty services like massed PTSD treatment. Although guidelines exist for some types of outpatient care in behavioral health, they are preliminary and have not been widely adopted.^35^

Through the implementation of continual and rigorous program evaluation, the authors found evidence that the formation of a small, dedicated intake team formed for the specific purpose of completing intakes not only decreased wait times but also reduced dropout. Reducing dropout has clear implications for clinic administrators, given that reduced dropout may mean the ability to serve more patients. Forming this team improved staff role clarity, supported consistent performance in implementing clinic procedures, and facilitated measurement of the intake process. Despite overall improvements in clinic performance, some inequities regarding demographic variables persisted, highlighting the need to pay special attention to the barriers and mental health needs of these groups specifically.

## Data Availability

The dataset used in the current study is not publicly available. The dataset can be obtained from the corresponding author upon reasonable request.

## Footnotes

1 Interactions were explored, but they were not significant.
