# Correction to: Implementation of a Dedicated Intake Team Reduces Time to Massed PTSD Treatment

**DOI:** 10.1007/s11414-025-09931-9

**Published:** 2025-01-31

**Authors:** Jennifer A. Coleman, Brianna Werner, Brian J. Klassen, Dale L. Smith, Uddyalok Banerjee, Philip Held

**Affiliations:** 1 Department of Psychiatry, Rush University Medical Center, 1645 W. Jackson Blvd., Suite 602, Chicago, IL 60612 USA; 2 Department of Psychiatry, Rush University Medical Center, Chicago, IL 60612 USA; 3 Department of Psychiatry, University of Illinois at Chicago, Chicago, IL USA

**Correction to: The Journal of Behavioral Health Services & Research**

10.1007/s11414-024-09920-4

The abstract was inadvertently left out of the published article. The original article has been corrected.

